# DSM-5 personality trait facets amongst child molesters: an exploratory comparison with other types of offenders

**DOI:** 10.1186/s40359-021-00619-1

**Published:** 2021-08-06

**Authors:** Fabio Ferretti, Felice Carabellese, Roberto Catanesi, Anna Coluccia, Stefano Ferracuti, Adriano Schimmenti, Vincenzo Caretti, Lore Lorenzi, Giacomo Gualtieri, Fulvio Carabellese, Andrea Pozza

**Affiliations:** 1 Department of Medical Sciences, Surgery and Neurosciences, University of Siena, Viale Mario Bracci 16, 53100 Siena, Italy; 2 Interdisciplinary Department of Medicine, Section of Criminology and Forensic Psychiatry, University of Bari “Aldo Moro”, Bari, Italy; 3 Department of Human Neuroscience, Sapienza University of Rome, Rome, Italy; 4 Faculty of Human and Social Sciences, UKE—Kore University of Enna, Enna, Italy; 5 Department of Human Sciences, LUMSA University of Rome, Rome, Italy

**Keywords:** Personality, Child molesters, DSM-5, PID5, Psychopathy

## Abstract

**Background:**

DSM-5 provided a dimensional model of personality disorders which may be more clinically informative for the assessment and management of prisoners than a categorical one, as diagnoses of personality disorders alone cannot explain the type of violence. The role of DSM-5 personality facets is however understudied in child molesters, and no study compared these clinical features between individuals who have committed sex crime against children and those who have committed other types of crime. The present study compared DSM-5 personality trait facets between prisoners who had committed sex crime against children, prisoners who had committed property crime (i.e., robbery, fraud) and those who had committed crime against the person (i.e., homicide, assault or violence not implying a sexual element). A further aim was to explore which facets were associated with sex crime against children as compared with the other types of crime, controlling for socio-demographics (age, gender), psychiatric comorbidity (presence of any psychiatric diagnoses) and general psychopathy traits.

**Methods:**

One hundred sixty-seven prisoners participated (91 had committed sex crime against children, 25 property crime, and 51 committed a crime against the person) and completed the Personality Inventory for the DSM-5 and the Psychopathy Checklist-Revised.

**Results:**

Prisoners who had committed sex crime against children reported higher Restricted Affectivity traits than those who had committed property crime and crime against the person and higher Irresponsibility traits than those who had committed property crime. The results of a multinomial logistic regression analysis showed that on the one hand being a man, having a higher age, and the presence of a psychiatric comorbidity were more likely to be related to sex crime than property crime, on the other hand higher Irresponsibility personality traits, being a man, and the presence of a psychiatric comorbidity were more likely to be related to sex crime against children than crime against the person.

**Conclusions:**

The Irresponsibility facet might be specific to child molesters and can differentiate this group from offenders who have committed other crime types. This facet might be considered a key target of a tailored assessment and treatment planning during clinical practice with child molesters.

## Background

### Beyond a categorical model in the personality assessment of child molesters: DSM-5 facets

It is well-established that personality pathology represents an individual vulnerability factor which can predict an increased risk for any type of crime [1–3]. Prevalence rates of diagnoses of personality disorders range between 42 and 78% in prisoners (e.g., [4]), being much higher than the rates usually observed in the general population, i.e., 4–13% [2, 5]. DSM Axis II disorders most frequently diagnosed in forensic populations and reported to be associated with any type of crime include antisocial, paranoid, borderline, and narcissistic personality disorders (e.g., [6, 7]).

The relationship between personality and crime types is under-studied. Research which assessed personality disorders through a categorical approach produced heterogenous, inconsistent evidence. The few studies found that any personality disorders were related to an increased likelihood of crime against the person and against the property [8], while other evidence showed that antisocial personality disorder was the most prevalent in prisoners who have committed such crimes [4, 8]. Other studies reported that certain personality disorders would be related to sex crime, particularly cluster B personality disorders [9, 10] and more specifically antisocial and borderline personality disorders [11]. However, most of the studies used only a group of prisoners who had committed a specific type of crime, but they did not compare personality pathology across different types of crime in the same study sample.

Overall, the available findings suggest that a categorical approach to personality is not able to differentiate between subgroups of offenders based on the crime type [12]. Some research focused on personality traits related to emotion regulation and showed similarities between child molesters and non-sexual violent offenders: both groups would have difficulty identifying the emotional expressions of others, would experience negative affective states (particularly anger and shame) and emotion dysregulation [13–15]. Other studies found that while sexual offenders would have circumscribed difficulties in emotional awareness and regulation, non-sexual violent offenders would experience more generalized problems in such personality traits [16].

The nature of the Diagnostic and Statistical Manual of Mental Disorders (DSM) categorial personality classification (e.g., [17]) has frequently been criticized because it lacks a cohesive, prototypical hierarchy of characteristics, gives equal weight to behaviourally based criteria that may be less central to the personality disorder they define. In addition, such a classification seems to result in high rates of comorbidity due to significant overlap between personality disorder diagnostic criteria [18–21]. Moreover, it is associated with low inter-rater agreement, and also it provides inadequate coverage of the range of personality disorder symptoms [18–20]. These methodological shortcomings can significantly hamper the precise characterization of the personality disorder–crime type relationship and limit the development of focused, tailored treatment approaches. Indeed, a growing body of evidence suggests that 60–70% offenders present psychopathological issues not fulfilling the diagnostic criteria of full personality disorders and therefore do not receive an adequate, tailored psychiatric or psychotherapeutic help [22].

To overcome the difficulties related to the previous versions of DSM personality diagnoses, the new classification of DSM-5 [23] provided an alternative, dimensional model of personality disorders in Section III, the area of the manual which includes also assessment measures, guidance on cultural formulation, and conditions for further study. A key aspect is that it offers an empirically based model of maladaptive personality domains and traits, which synthesizes existing dimensional models of personality dysfunctions focused on maladaptive variants. The model comprises five major domains of maladaptive personality: (1) Negative Affectivity (frequent and intense experiences of high levels of a wide range of negative emotions), (2) Detachment (avoidance of socio-emotional experience), (3) Antagonism (behaviours that put the individual at odds with other people), (4) Disinhibition (orientation toward immediate gratification and impulsive behaviour), and (5) Psychoticism (a wide range of culturally incongruent odd, eccentric, or unusual behaviours and cognition). These domains are articulated into 25 lower-order specific maladaptive personality traits (i.e., facets) which represent specific aspects of each general maladaptive domain [24]. This hierarchical model is similar to five-factor models (e.g., [25]); however, a key difference is the fact that five-factor models identify normal levels of personality traits, while DSM-5 facets capture abnormal ranges of personality dimensions found in personality disorders [24]. An overview of the personality domains and facets proposed in DSM-5 is presented in Table 1.

**Table 1:** Classification of personality domains and facets according to PID5

Domains	Facets
Negative affect	Separation insecurity
	Anxiousness
	Emotional lability
	Hostility
	Submissiveness
	Restricted affectivity
	Perseveration
Psychoticism	Unusual beliefs and experiences
	Perceptual dysregulation
	Eccentricity
Detachment	Anhedonia
	Intimacy avoidance
	Withdrawal
	Suspiciousness
	Depressivity
Antagonism	Grandiosity
	Manipulativeness
	Deceitfulness
	Attention seeking
	Callousness
Disinhibition	Irresponsibility
	Impulsivity
	Rigid perfectionism
	Risk taking
	Distractibility

*PID5* Personality Inventory for DSM5

The ability of instruments based on this new model (e.g., Personality Inventory for DSM-5; PID5; [26]) to assess extreme ranges of personality has proved valuable in several clinical contexts, and it is superior to instruments assessing aspects of specific personality disorders, such as the Narcissistic Personality Inventory (NPI; [27]). A key advantage of this new model is that personality disorders can be conceptualized in terms of specific constellations of maladaptive traits, rather than being distinct constructs from each other and from normal personality [28]. The dimensional model has demonstrated improved clinical utility of the personality assessment, particularly in orienting prognostic judgement and treatment decision making [29]. For example, in a national sample of 337 clinicians who were asked to provide complete personality disorder diagnostic information and several treatment-related clinical judgments about one of their patients, this model of personality predicted clinicians' decisions better than did the DSM-IV categories in 10 of the 11 studied clinical judgments of treatment-related decision-making [30].

There is a paucity of studies which investigated the association between DSM-5 personality trait facets and crime, particularly across different types of crime. Recent evidence showed that specific DSM-5 personality facets (i.e., Hostility, Risk Taking, Impulsiveness, Manipulativeness, Deceitfulness) were associated with a history of crime (e.g., [31–33]). Other studies showed that Callousness, Grandiosity, Impulsiveness, and Risk Taking were predictive of both psychopathy- and narcissism-related traits, which are typically present among prisoners [34, 35].

Adhiatma and Halim [36] found that as compared with a non-prisoner group, prisoners who had committed crime against the person had higher levels on a number of facets including Hostility, Depressivity, Suspiciousness, Callousness, Withdrawal, Intimacy Avoidance, Anhedonia, Restricted Affectivity, Irresponsibility, Impulsiveness, Unusual Beliefs and Experiences, and Perceptual Dysregulation. In addition, as compared with a non-prisoner group, prisoners who had committed property crime had higher Depressivity, Suspiciousness, Callousness, Withdrawal, Intimacy Avoidance, Anhedonia, Restricted Affectivity, Unusual Beliefs and Experiences, and Perceptual Dysregulation [36]. In a more recent study using the PID-5, Russell and King [31] found that Suspiciousness, Cognitive and Perceptual Dysregulation, Grandiosity, and lack of Eccentricity emerged as predictors of sexual violence. In conclusion, the few studies provided heterogenous evidence on the relation between DSM-5 facets and crime, and little is known about the role of such traits across different crime types.

### Rationale and objectives

The role of DSM-5 personality facets is understudied in child molesters, and no study compared the personality facets between individuals who have committed sex crime against children and those who have committed other types of crime such as crime against the person and the property.

The first aim of the present study was to compare DSM-5 personality trait facets between prisoners who had committed sex crime against children, prisoners who had committed property crime and those who had committed crime against the person (i.e., homicide, assault or violence not implying a sexual element). The second aim was to investigate which specific DSM-5 personality facets were associated with sex crime against children as compared with the other types of crime, controlling for socio-demographics (age, gender), psychiatric comorbidity (presence of any psychiatric diagnoses according to DSM-5) and general psychopathy traits which are variables already found to be related to crime. According to the literature evidence (e.g., [6, 12, 31, 37]), we hypothesized that (a) psychopathy traits are related to a higher likelihood of having committed a property crime or crime against the person than sex crime against children, (b) being a man is related to a higher likelihood of having committed a sex crime against children than a property crime or crime against the person. With respect to the specific role of the personality facets in sex crime against children, we had not specific hypotheses since this is the first study on this topic and we explored the specificity of the personality facets in this group of prisoners.

## Methods

### Procedure and eligibility criteria

This study is part of a large national multicentre research project [38] approved and authorized by the Penitentiary Administration Department of the Ministry of Justice in accordance with the ethical standards identified and in compliance with the privacy rules on sexual crimes. The multicentre project was conducted in the prisons of six Italian regions where the university centre coordinator of the project guaranteed the uniformity and homogeneity of the data collection. All the researchers involved had been trained through role-playing and internships to administer the tools before the research was initiated.

The investigation was carried out from 2015 to 2016. Each director of the prisons provided the lists of child molesters who had given consent to participate. A meeting with the prisoners and social workers was carried out to present the project and obtain participants’ written informed consent [39]. The subjects involved were informed about the purpose of the research and provided their written informed consent.

The collection of the anamnestic and criminological data was conducted by analysing the clinical records through an ad-hoc module. All data were processed anonymously.

To be included, the subjects had to be prisoners who had received a final conviction for only a specific crime type. Three groups were created: (a) child molesters (as defined in the conceptualization of Myers and colleagues [40]), (b) offenders who had committed a crime against the person (e.g., homicide, assault not implying a sexual element), (c) prisoners who had committed a crime against the property (i.e., offenses committed against the property of other people; e.g., robbery, fraud).

### Measures

#### Personality Inventory for the DSM-5 (PID-5 [23])

The PID-5 is a 220-item, self-report instrument developed [23] to assess trait facets and domains. Items are rated on a 4-point Likert-type scale ranging from 0 (very false or often false) to 3 (very true or often true). Scoring for the PID-5 generates the five trait domain scales and 25 lower order facet scales. In this study the focus was on the facet scales. The Italian version of the PID-5 Informant Report [41] was adopted to determine the presence of major psychiatric disorders and personality facets.

#### Psychopathy Checklist-Revised (PCL-R; [42])

The PCL-R measures psychopathic personality traits. It includes 20 items (scored 0, 1 or 2), which are rated based on an interview with the participant and information from clinical records.

A version of the PCL-R validated for the Italian population [43] was administered to all the subjects. A threshold score equal to or greater than 25 was established to identify the condition of psychopathy, as indicated in studies conducted in European populations [44–46] and as applied in our previous studies [47–49].

### Statistical analyses

Differences between the three groups on socio-demographic (age and gender) and clinical features (personality facets, presence of any type of psychiatric comorbidity, and general psychopathy traits as indicated by a PCL-R score higher than 25) were calculated through non-parametric tests for dichotomous variables and parametric analyses (ANOVAs with Scheffé post-hoc comparisons) for continuous variables, respectively. Effect sizes calculated as squared eta (*η*^2^) of 0.01, 0.06, 0.14 were interpreted as small, medium, and large, respectively [50].

An a-priori power analysis suggested that the required sample size necessary to detect a medium effect with 80% power and a *p*-value of 0.05 was 159 subjects.

Subsequently, we investigated the effects of the personality facets on crime type controlling for gender, age, psychiatric comorbidity (any type of psychiatric diagnosis according to the DSM-5), and psychopathy traits (a score on the PCL-R higher than 25) by performing a multinomial logistic regression analysis. The statistical significance for this analysis was set at *p* < 0.05. The statistical analyses were carried out through the SPSS software version 23.

## Results

### Descriptive characteristics of the groups

One hundred sixty-seven prisoners were included, of whom 91 had committed sex crime against children, 25 property crime, and 51 committed a crime against the person. The demographic and clinical features of the three groups are presented in Table 2. The three groups were significantly different on age (sex offender group was older than the other two groups), on gender distribution (the number of men was higher in the child molester group than in those who have committed crime against the person where it was in turn higher than the property crime group), and psychiatric comorbidity (the number of prisoners having DSM-5 psychiatric comorbidity was higher among crime against the person group than the property crime group where it was higher than the child molester group). No difference emerged on the levels of psychopathy traits as indicated by a score higher than 25 on the PCL-R.

**Table 2:** Socio-demographic and clinical characteristics of the groups

	Sex crime against children (*n* = 91)		Property crime (*n* = 25)		Crime against the person (*n* = 51)		*F/*Kruskal–Wallis H test	*p*-value	Post-hoc comparison
	*n* (%)	Mean (SD; range)	*n* (%)	Mean (SD; range)	*n* (%)	Mean (SD; range)			
Age (years)		48.78 (13.23; 22–75)		39.40 (9.30; 26–55)		42.53 (10.85; 21–81)	8.15	< 0.001	Sex child crime > property crime = crime against the person
Gender							51.51	< .001	Sex child crime > crime against the person > property crime
Men	88 (96.70)		9 (36)		42 (82.40)				
Women	3 (3.30)		16 (64)		9 (17.60)				
Psychiatric comorbidity							64.93	< 0.001	Crime against the person > property crime > sex child crime
No	84 (92.30)		12 (48)		14 (27.50)				
Yes	7 (7.70)		13 (52)		37 (72.50)				
Type of psychiatric comorbidity									
Unipolar depressive disorders	3 (3.30)		1 (4)		1 (2)				
Bipolar disorders	1 (1.10)		2 (8)		6 (11.80)				
Psychotic disorders	2 (2.20)		0		25 (49)				
Substance abuse disorder	0		7 (28)		2 (3.90)				
Personality disorders	1 (1.10)		3 (12)		3 (5.90)				
Psychopathy traits (PCL-R > 25)							0.20	0.90	
Yes	10 (11)		2 (8)		5 (9.80)				
No	81 (89)		23 (92)		46 (90.20)				

*PCL-R* psychopathy checklist revised

### Differences on PID5 facets between types of crime

The results of the ANOVAs (Table 3) showed no significant differences on all the domains measured by the PID5 between the three groups.

**Table 3:** Comparisons between groups on the PID5 domains and facets

	Sex crime against children (*n* = 91)		Property crime (*n* = 25)		Crime against the person (*n* = 51)		*F*_(2, 157)_	*p-value*	*η*^*2*^	
	Mean	SD	Mean	SD	Mean	SD				
PID5 domains										
PID5 negative affectivity	1.165	0.675	1.107	0.635	1.192	0.706	0.125	0.883	0.002	
PID5 detachment	0.919	0.635	0.699	0.457	0.844	0.484	1.448	0.238	0.018	
PID5 antagonism	0.597	0.586	0.684	0.555	0.514	0.491	0.782	0.459	0.010	
PID5 disinhibition	0.895	0.713	0.863	0.455	0.715	0.527	0.771	0.464	0.010	
PID5 psychoticism	0.629	0.637	0.672	0.496	0.377	0.584	2.085	0.128	0.026	

	Sex crime against children (*n* = 91)		Property crime (*n* = 25)		Crime against the person (*n* = 51)		*F*_(2, 157)_	*p-value*	*η*^*2*^	Scheffé post-hoc test
	Mean	SD	Mean	SD	Mean	SD				
PID5 facets										
PID5—restricted affectivity	0.86	0.60	0.45	0.53	0.96	0.62	6.13	0.003	0.073	Crime against the person > sex child crime > property crime
PID5—anhedonia	0.81	0.57	0.72	0.49	0.83	0.46	0.38	0.68	0.005	
PID5—separation insecurity	0.86	0.70	0.82	0.70	1.020	0.83	0.87	0.42	0.011	
PID5—anxiousness	1.35	0.97	1.34	0.68	1.39	0.72	0.04	0.95	0.001	
PID5—unusual beliefs and experiences	0.68	0.84	0.39	0.58	0.75	0.64	1.91	0.15	0.024	
PID5—depressivity	0.81	0.83	0.88	0.69	0.75	0.59	0.26	0.76	0.003	
PID5—perceptual dysregulation	0.49	0.65	0.33	0.51	0.60	0.61	1.58	0.20	0.020	
PID5—distractibility	0.80	0.64	0.79	0.63	0.83	0.68	0.02	0.97	0.000	
PID5—eccentricity	0.68	0.62	0.48	0.65	0.76	0.76	1.36	0.25	0.017	
PID5—intimacy avoidance	0.85	1.04	0.46	0.49	0.68	0.63	1.99	0.14	0.025	
PID5—grandiosity	0.60	0.56	0.59	0.63	0.80	0.77	1.73	0.17	0.022	
PID5—impulsivity	0.90	0.77	0.84	0.89	0.97	0.78	0.23	0.78	0.003	
PID5—deceitfulness	0.45	0.47	0.47	0.51	0.58	0.57	0.98	0.37	0.012	
PID5—callousness	0.46	0.43	0.36	0.42	0.56	0.56	1.61	0.20	0.020	
PID5—irresponsibility	0.72	0.59	0.41	0.36	0.61	0.48	3.27	0.04	0.040	Sex child crime > property crime
PID5—emotional lability	1.07	0.71	1.19	0.81	1.22	0.86	0.63	0.53	0.008	
PID5—manipulativeness	0.51	0.58	0.47	0.52	0.75	0.64	3.09	0.05	0.038	
PID5—hostility	0.80	0.64	0.96	0.86	0.89	0.55	0.67	0.51	0.009	
PID5—rigid perfectionism	1.25	0.65	1.27	0.86	1.28	0.74	0.02	0.97	0.000	
PID5—perseveration	0.89	0.54	0.73	0.58	0.94	0.67	1.10	0.33	0.014	
PID5—attention seeking	0.76	0.82	0.71	0.80	0.94	0.79	1.13	0.32	0.014	
PID5—withdrawal	1.02	0.06	0.92	0.80	0.98	0.62	0.23	0.78	0.003	
PID5—suspiciousness	1.20	0.63	1.18	0.62	1.19	0.61	0.01	0.98	0.000	
PID5—submissiveness	0.74	0.69	0.81	0.76	0.86	0.70	0.50	0.60	0.006	
PID5—risk taking	1.04	0.63	1.02	0.58	1.22	0.61	1.48	0.23	0.019	

*PID5* Personality Inventory for DSM-5

A significant difference on the PID5 Restricted Affectivity scores emerged across crime types with a medium effect size: prisoners who had committed a sex crime against children had significantly higher scores on the PID5 Restricted Affectivity than those who had committed a property crime and a crime against the person. Prisoners who had committed a crime against the person had significantly higher scores than those who had committed a property crime. When a Bonferroni correction for multiple tests (*p* = 0.05/30 = 0.0016) was applied, this difference became not significant.

In addition, a significant difference emerged on the PID5 Irresponsibility scores across crime types with a small effect size: prisoners who had committed a sex crime had significantly higher PID5 Irresponsibility scores than those who had committed a property crime. No significant differences emerged between the groups on the other PID5 facets. When a Bonferroni correction for multiple tests (*p* = 0.05/30 = 0.0016) was applied, this difference became not significant.

### Effects of PID5 facets on crime type controlling for demographic and clinical features

The results of the multinomial logistic regression analysis (Table 4) suggested that being a man, having a higher age, and the presence of a psychiatric comorbidity were more likely to be related to sex crime against children than property crime. Being a man, having higher scores on the PID5—Irresponsibility, and the presence of a psychiatric comorbidity were more likely to be related to sex crime against children than crime against the person.

**Table 4:** Multinomial logistic regression analysis of type of crime (reference category: sex crime against children)

Type of crime	*B*	Wald test	*df*	*p*-value
Property crime				
Intercept	5.98	8.12	1	0.004
Gender				
Male	− 3.79	20.38	1	0.000
Female	0^a^		0	
Age (years)	− 0.72	5.44	1	0.020
PID5—restricted affectivity	− 0.60	0.84	1	0.357
PID5—irresponsibility	− 1.17	1.90	1	0.168
Psychiatric comorbidity (Yes)	2.32	9.95	1	0.002
Psychopathy traits (PCL-R > 25)	− 0.78	0.349	1	0.555
Crime against the person				
Intercept	1.50	0.80	1	0.370
Gender				
Male	− 1.79	4.55	1	0.033
Female	0^a^		0	
Age (years)	− 0.03	3.08	1	0.079
PID5—restricted affectivity	0.68	2.22	1	0.136
PID5—irresponsibility	− 1.37	5.72	1	0.017
Psychiatric comorbidity (yes)	3.32	34.28	1	0.000
Psychopathy traits (PCL-R > 25)	0.27	0.09	1	0.761

*PCL-R* Psychopathy Checklist Revised, *PID5* Personality Inventory for DSM5

^a^This parameter is set at 0 because redundant in the model

## Discussion

### Main findings

The present study is the first investigation which explored DSM-5 personality trait facets in prisoners who had committed different types of crime, specifically those who had committed sex crime against children compared with prisoners who had committed property crime and prisoners who had committed crime against the person.

The results of multinomial logistic regression analyses showed that prisoners who were men, had an older age and a psychiatric comorbidity were more likely to be child molesters as compared with prisoners who have committed a property crime, while none of the personality facets differentiated between these two groups. Prisoners who were men, had a psychiatric comorbidity and a higher irresponsibility facet were more likely to be child molesters than non-sexual violent offenders.

The association between gender and sex crime is in line with our hypothesis and with literature data showing that amongst men the probability of having committed a sex crime against children would be higher as compared with other types of crime (e.g., [31, 37–39]). The effect of age may be due to detection rates since it takes more time until a child molester gets detected and convicted compared to the other subgroups, as pedophilic crimes happen regularly in the context of families and are therefore more difficult to detect [51].

Being a man, the presence of a psychiatric comorbidity, and a higher irresponsibility facet were more likely to be related to sex crime against children as compared with crime against the person. The role of gender appears consistent with some general aspects of the Dual Control Model which assumes that men would have a higher propensity to sexual excitation and a lower propensity to sexual inhibition than women [52, 53].

The specific role of irresponsibility may be considered in line with evidence showing the role of moral disengagement in sex crime against children (i.e., the process of convincing the self that ethical standards do not apply to oneself in a particular context) [54] and with the theoretical considerations of Mann and Marshall [55] who suggested that taking responsibility for their offending should be considered as one of the core elements of the treatment approaches for child molesters. Irresponsibility might act as a trait facilitator of child sexual offending. Consistent with this hypothesis, according to the Motivation-Facilitation Model by Seto [56], primary motivations for sexual offenses (e.g., pedophilic traits, high sex drive, and intense mating effort), as well as trait (e.g., antisocial personality traits) and state (e.g., intoxication states) factors can facilitate acting on these motivations when situational opportunities exist.

In addition, the key role of irresponsibility might be considered consistent with the widely reported evidence that antisocial personality disorder is the most prevalent personality disorder among child molesters [11]. The specific association between irresponsibility and sex crime against children seems to be consistent also with previously published data based on the five-factor model which showed that child molesters report low levels of conscientiousness, an opposite construct to irresponsibility [57].

The role of the irresponsibility personality facet should be considered in the context of the so-called stable dynamic risk factors involved the relapses of child molesters, i.e., relatively enduring psychological or behavioural features of the offenders that raise the risk of reoffending and that are potentially changeable [58]. The role of the irresponsibility facet may be regarded consistent with the evidence coming from meta-analytic studies which suggested that general self-regulation problems and resistance to rules are empirically supported stable dynamic risk factors involved in recidivism of sex offenders and lack of concern of others are promising stable dynamic risk factors [59].

The lack of a significant relation between psychopathy traits and property crime or crime against the person appears somewhat unexpected and in contrast with previous evidence indicating that these traits are more elevated amongst these types of prisoners than community adults [60–62]. It should be noted that the present study is the first one which compares the levels of psychopathy traits across different types of crime. However, the role of psychopathy traits has been found to be less specific to crime in the literature, and some data has brought into question the utility of this construct, particularly if used as unitary concept [63].

### Clinical implications for a tailored assessment and treatment planning

From a clinical perspective, irresponsibility should be considered as a key target of the assessment and tailored intervention/relapse prevention strategies for child molesters. A variety of promising strategies might be helpful to target this dysfunctional personality facet including group-based cognitive behavioural therapy including different components aimed to enhance taking the responsibility for the crime, empathy, compassion, self-forgiveness and mentalization [64–66]. For example, compassion-focused therapy techniques developed by Paul Gilbert might be a useful strategy for increasing the empathy skills of child molesters [67]. In addition, the strengths of group-based cognitive behavioural approaches should be considered, as they can reduce the risk of drop-out rates for several forms of psychopathologies to a greater extent than individual approaches (e.g., [68]).

The presence of a higher likelihood of psychiatric comorbidities in the child molester group suggests that clinicians should focus their attention also on this clinical feature during the assessment and treatment and that they should expect that the child molester group might present a more complex and severe clinical picture than the other groups. For example, the integration of the Structured Clinical Interview for DSM-5 Disorders-Clinician Version may be useful in the assessment of child molesters [69]. In addition, the present findings may be considered in the context of the literature on dynamic risk factors of recidivism and perhaps suggest that the PID5 Irresponsibility facet scale might be used and integrated into the so-called third-generation tools which are actuarial measures designed to assist intervention efforts that assess criminogenic needs [70].

The lack of a significant relation between psychopathy traits and property crime or crime against the person suggest that the construct of psychopathy as a unitary element might not be specific and clinically informative in the assessment of prisoners who have committed different types of crime.

### Additional findings

Analyses based on ANOVAs did not detect differences between the three groups on the personality domains, but they suggested differences on two specific personality facets. On the one hand, prisoners who had committed crime against the person had more elevated restricted affectivity than those who had committed sex crime against children and those who had committed property crime. On the other hand, child molesters reported higher restricted affectivity than prisoners who had committed property crime. The result related to high levels of restricted affectivity among child molesters may be viewed in line with theoretical perspectives and empirical evidence [71, 72] suggesting that as compared with the general population child molesters would have high alexithymia (i.e., difficulty identifying and verbalizing emotions), a construct similar to restricted affectivity. The role of alexithymia in coercive sexual behaviour and recidivism has also been demonstrated by Engel and colleagues [73]. It may be hypothesized that child molesters with alexithymic traits might use sexual coercive behaviours as a relational strategy to experience emotions by provoking strong negative emotions in other people. This suggests that interventions aimed to target restricted affectivity might be helpful specifically for prisoners who committed sex crime against children. Indeed, Byrne et al. [74] developed a treatment protocol with modules designed to increase the capacity to get in contact with, identify and verbalize emotions and the related body signals. The high levels of restricted affectivity might be in line also with the literature showing a lack of empathy levels amongst child molesters (e.g., [75, 76]) and might support the importance of providing this subgroup of prisoners with a therapeutic pathway focused on improving empathic skills [77]. It should be noted that the group of prisoners who had committed crime against the person had higher levels of restricted affectivity than the other two groups, in line with data indicating more generalized difficulties in emotional awareness and regulation amongst non-sexual violent offenders [14]. This suggested that also the group of prisoners who committed crime against the person might benefit from interventions aimed to target this dysfunctional personality trait. The high levels of restricted affectivity in those who have committed a crime against the person is consistent with other data showing that this group of offenders have higher alexithymia than child molesters [14].

In addition, we found that prisoners who had committed sex crime against children had more elevated irresponsibility than those who had committed property crime, in line with previous data based on other instruments than PID5 showing that this personality trait is high in this offender population [78].

In contrast with previous evidence [36], we did not detect a role of suspiciousness, cognitive and perceptual dysregulation, grandiosity, and a lack of eccentricity as predictors of sex crime. An explanation for this result might be the fact that in our statistical model we controlled for the effect of psychiatric comorbidity which had not been controlled for in previous studies. Indeed, in our study in the group of prisoners who committed crime against the person the prevalence of psychiatric comorbidities was higher than the property crime group where it was in turn higher than the child molester group. The above-mentioned personality traits were commonly found to be related to mood, personality, and psychotic spectrum disorders [79, 80].

The lack of differences in some trait facets such as separation insecurity, anxiousness, depressivity, emotional lability, hostility, and withdrawal is in line with previous data showing similarities in the personality profile of child molesters and non-sexual violent offenders since both groups present difficulty in identifying the emotional expressions of others, the experience of negative affective states (e.g., anger and shame) and emotion dysregulation among sexual and violent offenders [14, 15]. Overall, the present findings highlight the possibility that most of DSM5 personality traits are common to all three types of crime. Furthermore, the lack of a control group with people who have not committed any crime type did not allow us to verify whether the levels on some traits were higher than in a group without psychiatric disorders. Other studies however found that while sexual offenders had some circumscribed difficulties in emotional regulation, non-sexual violent offenders showed more generalized problems in such personality traits [16].

### Limitations and future directions

The cross-sectional design did not allow us to draw firm conclusions about the role of the personality facets as risk factors for sex crime against children. A bidirectional effect or even an inverse relation might be hypothesized: it may be speculated that having committed sex crime against children can lead to the development or even exacerbation of some personality facets in the phases after the crime, particularly if they are not timely assessed and appropriately targeted by effective interventions in the forensic setting [81].

Following a relapse prevention approach, it might be interesting to focus on the role of the personality facets as risk factors for recidivism. Future longitudinal studies should ascertain whether specific facets are risk factors of relapse on a specific type of crime.

Another limitation regards the use of self-report instruments. Future research should use additional modalities of assessment of personality such as clinician-administered interviews and psychophysiological measures. Moreover, the inclusion of a malingering measure might improve the reliability of the assessment procedure.

The relatively small size of the property crime group might have increased the likelihood of a type-II error due to the low statistical power. This point may be particularly important for Manipulativeness facets which showed a borderline *p*-value of 0.05. However, it should be noted that the ANOVA-based comparisons of the other facets which did not result significant were associated with a range of *p*-values of 0.14–0.98, very far from the chosen statistical threshold and the a-priori power analysis suggests that the present sample size was sufficient to detect a medium effect. In addition, the findings about a higher probability of having committed a sex crime against children amongst men as compared with the other two crime types should be considered more critically, as there was only a small comparable group of women involved in this study. It should be noted that the perpetration of sex crime against children by women is generally quite rare as compared with perpetration by men [82] and recent data suggest that child molesters who are men are more likely to be sentenced to prison, and given longer terms, than child molesters who are women [83, 84].

As previously mentioned, the absence of a control group did not allow us to verify whether, or not, these three groups had more elevated levels on some dysfunctional personality traits than normal personality functioning (i.e., in community or screened healthy samples). Indeed, it should be mentioned that the prevalence rates of any personality disorders in the general population ranges from 4 to 13% according to the most recent meta-analyses [2, 5, 85, 86].

The lack of an effect of psychopathy traits might be attributed to the low statistical power as well; however, it should be noted that in our sample, the 8–11% proportion of subjects with psychopathy traits are consistent with more recent evidence showing that psychopathy traits are present only amongst a subgroup of about 7% offenders [60]. It might be interesting to evaluate whether specific psychopathy traits would be more closely related to sex crime against children than high levels of psychopathy considered as a unitary concept.

The fact that most of the personality trait facets were not specific to any crime type does not necessarily suggest that they should not be assessed, but it only suggests that they might not be considered a specific target of the assessment and treatment for a specific group of offenders, and they should be considered in a transdiagnostic approach. Since our approach was based upon a comparison between different types of crime, another relevant point to be assessed in future studies might be the network structure of the personality facets across different types of crime by using the recently developed network approaches [87–89] with the aim to highlight the centrality of some personality facets. It would be useful to explore which personality facets influence each other in a dynamic inter-relationship instead of a static model that considers each one of them separately.

Another issue that deserves attention in future studies is the role of specific types of psychiatric comorbidities across different crime types; unfortunately, due to the small size of the subgroups in the present study, we were not able to explore this association. Another interesting aspect to be investigated regards the role of socio-cultural variables such as the immigrant status [90].

Finally, in future research other relevant features should be considered as potential covariates such as the duration of the imprisonment and the presence of specific personality disorders.

## Conclusions

This exploratory study is the first investigation which used the new dimensional model of DSM-5 to examine the maladaptive personality facets in child molesters as compared with other types of offenders. A first analysis showed the specific role of the irresponsibility and restricted affectivity facets in differentiating child molesters from prisoners who have committed other types of crime (crime against the person or the property).

A deeper examination showed that men, older individuals, and those having a psychiatric comorbidity were more likely to be child molesters as compared with prisoners who have committed a property crime, while none of the personality facets differentiated between these two groups. Men, those prisoners with a psychiatric comorbidity and those with a higher irresponsibility facet were more likely to be child molesters than non-sexual violent offenders. In conclusion, the irresponsibility facet might be considered a key target of a tailored assessment and treatment planning during clinical practice with child molesters.

## Data Availability

The datasets used and/or analysed during the current study are available from the corresponding author on reasonable request.

## Abbreviations

DSM: Diagnostic and Statistical Manual of Mental Disorders
PID5: Personality Inventory for DSM-5
NPI: Narcissistic Personality Inventory
PCL-R: Psychopathy Checklist-Revised

## References

[1] Blackburn R, Logan C, Donnelly J, Renwick S (2003). Personality disorders, psychopathy and other mental disorders: co-morbidity among patients at English and Scottish high-security hospitals. J Forensic Psychiatry Psychol.

[2] Coid J, Yang M, Tyrer P, Roberts A, Ullrich S (2006). Prevalence and correlates of personality disorder in Great Britain. Br J Psychiatry.

[3] Coluccia A, Pozza A, Ferretti F, Carabellese F, Masti A, Gualtieri G (2020). Online romance scams: relational dynamics and psychological characteristics of the victims and scammers. A scoping review. Clin Pract Epidemiol Ment Health.

[4] Fazel S, Danesh J (2002). Serious mental disorder in 23 000 prisoners: a systematic review of 62 surveys. Lancet.

[5] Torgersen S, Kringlen E, Cramer V (2001). The prevalence of personality disorders in a community sample. Arch Gen Psychiatry.

[6] Flórez G, Ferrer V, García LS, Crespo MR, Pérez M, Saiz PA (2019). Personality disorders, addictions and psychopathy as predictors of criminal behaviour in a prison sample. Revista espanola de sanidad penitenciaria.

[7] Mazzoni GP, Contena B, Fanciullacci S, Pozza A (2018). Analisi comparativa dei profili di personalità di pazienti affetti da disturbo borderline e detenuti con diagnosi di disturbo antisociale di personalità ottenuti tramite l’MMPI-2. Riv Psichiatr.

[8] Black DW, Gunter T, Loveless P, Allen J, Sieleni B (2010). Antisocial personality disorder in incarcerated offenders: psychiatric comorbidity and quality of life. Ann Clin Psychiatry.

[9] Borchard B, Gnoth A, Schulz W (2003). Personality disorders and “psychopathy” in child molesters imprisoned in forensic-psychiatric hospitals–SKID-II-and PCL-R-results in patients with impulse control disorder and paraphilia. Psychiatr Prax.

[10] Eher R, Rettenberger M, Turner D (2019). The prevalence of mental disorders in incarcerated contact sexual offenders. Acta Psychiatr Scand.

[11] Chen YY, Chen CY, Hung DL (2016). Assessment of psychiatric disorders among child molesters: prevalence and associations with criminal history. Crim Behav Ment Health.

[12] Ferracuti S, Mandarelli G, Del Casale A, Carpiniello B, Vita A, Mencacci C (2020). Psychopathy, personality disorders, and violence. Violence and mental disorders.

[13] Gillespie SM, Mitchell IJ, Fisher D, Beech AR (2012). Treating disturbed emotional regulation in sexual offenders: the potential applications of mindful self-regulation and controlled breathing techniques. Aggress Violent Behav.

[14] Roberton T, Daffern M, Bucks RS (2014). Maladaptive emotion regulation and aggression in adult offenders. Psychol Crime Law.

[15] Velotti P, Garofalo C, Callea A, Bucks RS, Roberton T, Daffern M (2017). Exploring anger among offenders: the role of emotion dysregulation and alexithymia. Psychiatr Psychol Law.

[16] Gillespie SM, Garofalo C, Velotti P (2018). Emotion regulation, mindfulness, and alexithymia: Specific or general impairments in sexual, violent, and homicide offenders?. J Crim Justice.

[17] American Psychiatric Association. Diagnostic and statistical manual of mental disorders (4th edn. Text revision). American Psychiatric Association; 2000.

[18] Gilbert F, Daffern M (2011). Illuminating the relationship between personality disorder and violence: contributions of the General Aggression Model. Psychol Violence.

[19] McGlashan TH, Grilo CM, Sanislow CA, Ralevski E, Morey LC, Gunderson JG (2005). Two-year prevalence and stability of individual DSM-IV criteria for schizotypal, borderline, avoidant, and obsessive-compulsive personality disorders: toward a hybrid model of axis II disorders. Am J Psychiatry.

[20] Verheul R, Widiger TA (2004). A meta-analysis of the prevalence and usage of the personality disorder not otherwise specified (PDNOS) diagnosis. J Pers Disord.

[21] Pozza A, Starcevic V, Ferretti F, Pedani C, Crispino R, Governi G (2021). Obsessive-compulsive personality disorder co-occurring in individuals with obsessive-compulsive disorder: a systematic review and meta-analysis. Harv Rev Psychiatry.

[22] Bulten E, Nijman H, van der Staak C (2009). Psychiatric disorders and personality characteristics of prisoners at regular prison wards. Int J Law Psychiatry.

[23] American Psychiatric Association (2013). Diagnostic and statistical manual of mental disorders (DSM-5®).

[24] Krueger RF, Markon KE (2014). The role of the DSM-5 personality trait model in moving toward a quantitative and empirically based approach to classifying personality and psychopathology. Ann Rev Clin Psychol.

[25] Costa PT, McCrae RR (1990). Personality disorders and the five-factor model of personality. J Pers Disord.

[26] Krueger RF, Derringer J, Markon KE, Watson D, Skodol AE (2012). Initial con-struction of a maladaptive personality trait model and inventory for DSM-5. Psychol Med.

[27] Raskin R, Hall CS (1981). The Narcissistic Personality Inventory: alternative form reliability and further evidence of construct validity. J Pers Assess.

[28] Hopwood CJ, Wright AG, Ansell EB, Pincus AL (2013). The interpersonal core of personality pathology. J Pers Disord.

[29] Morey LC, Archer RP, Smith SR (2014). The personality assessment inventory. Personality assessment.

[30] Dunne AL, Gilbert F, Daffern M (2018). Investigating the relationship between DSM-5 personality disorder domains and facets and aggression in an offender population using the personality inventory for the DSM-5. J Pers Disord.

[31] Russell TD, King AR (2020). Distrustful, conventional, entitled, and dysregulated: PID-5 personality facets predict hostile masculinity and sexual violence in community men. J Interpers Violence.

[32] Wygant DB, Sellbom M, Sleep CE, Wall TD, Applegate KC, Krueger RF (2016). Examining the DSM-5 alternative personality disorder model operationalization of antisocial personality disorder and psychopathy in a male correctional sample. Pers Disord Theory Res Treat.

[33] Morey LC, Benson KT (2016). Relating DSM-5 section II and section III personality disorder diagnostic classification systems to treatment planning. Compr Psychiatry.

[34] Anderson JL, Sellbom M, Wygant DB, Salekin RT, Krueger RF (2014). Examining the associations between DSM-5 section III antisocial personality disorder traits and psychopathy in community and university samples. J Pers Disord.

[35] Wright AG, Pincus AL, Thomas KM, Hopwood CJ, Markon KE, Krueger RF (2013). Conceptions of narcissism and the DSM-5 pathological personality traits. Assessment.

[36] Adhiatma W, Halim MS (2016). Personality profile differences between prisoners and non-prisoners using the personality inventory for DSM-5 (PID-5). ANIMA Indones Psychol J..

[37] Di Cosimo D, Ferracuti S (2013). Le differenze di genere nella genetica dei comportamenti aggressivi. Ital J Criminol.

[38] Caretti V, Ciappi S, Scarpa F, Castelletti L, Catanesi R, Carabellese F, Ferracuti S, Nava F, Nicolò G, Paterniti R, Rivellini G, Schimmenti F (2019). HCR 20–3 Checklist per la valutazione del rischio di recidiva di un crimine violento. Adattamento italiano.

[39] Mandarelli G, Carabellese F, Parmigiani G, Bernardini F, Pauselli L, Quartesan R, Catanesi R, Ferracuti S (2017). Treatment decision-making capacity in non-consensual psychiatric treatment: a multicentre study. Epidemiol Psychiatr Sci.

[40] Myers WC, Marrero L, Herkov MJ, Payne-James J (2005). Forensic psychiatry and forensic psychology. Encyclopedia of forensic and legal medicine.

[41] Fossati A, Borroni S, Somma A (2015). Scale di valutazione PID-5.

[42] Hare RD, Neumann CS. The PCL-R assessment of psychopathy: Development structural properties, and new directions. In: Patrick C (ed.) Handbook of psychopathy. New York: The Guilford Press; 2006. p. 58–90.

[43] Caretti V, Manzi GS, Schimmenti A, Seragusa L (2011). PCL-R. Hare psychopathy checklist revised.

[44] Strand S, Belfrage H (2005). Gender differences in psychopathy in a Swedish offender sample. Behav Sci Law.

[45] Hicks BM, Vaidyawathan U, Patrick CJ (2010). Validating female psychopathy subtypes: differences in personality, antisocial and violent behaviour, substance abuse, trauma, and mental health. Pers Dis.

[46] Kreis Mette KF, Cooke DJ (2011). Capturing the psychopathic female: a prototypicality analysis of the comprehensive assessment of psychopathic personality (CAPP) across gender. Behav Sci Law.

[47] Carabellese F, Felthous AR, Rossetto I, La Tegola D, Franconi F, Catanesi R (2018). Female residents with psychopathy in a high-security Italian hospital. J Am Acad Psychiatry Law.

[48] Carabellese F, Felthous AR, La Tegola D, Rossetto I, Montalbò D, Franconi F, Catanesi R (2019). Psychopathy and female gender: phenotypic expression and comorbidity: a study comparing a sample of women hospitalized in maximun security facility with women who were criminally sentenced and inprisoned. J Forensic Sci.

[49] Carabellese F, Felthous AR, La Tegola D, Rossetto I, Franconi F, Lucchini G, Catanesi R (2020). Female psychopathy: a descriptive national study of socially dangerous NGRI female offenders. Int J Law Psychiatry.

[50] Olejnik S, Algina J (2003). Generalized eta and omega squared statistics: measures of effect sizes for some common research designs. Psychol Methods.

[51] Pipe ME, Lamb ME, Orbach Y, Cederborg AC (2013). Child sexual abuse: disclosure, delay, and denial.

[52] Gomes ALQ, Janssen E, Santos-Iglesias P, Pinto-Gouveia J, Fonseca LM, Nobre PJ (2018). Validation of the sexual inhibition and sexual excitation scales (SIS/SES) in Portugal: assessing gender differences and predictors of sexual functioning. Arch Sex Behav.

[53] Janssen E, Vorst H, Finn P, Bancroft J (2002). The sexual inhibition (SIS) and sexual excitation (SES) scales: II. Predicting psychophysiological response patterns. J Sex Res.

[54] Petruccelli I, Simonelli C, Barbaranelli C, Grilli S, Tripodi MF, D'Urso G (2017). Moral disengagement strategies in child molesters. Psychiatr Psychol Law.

[55] Mann RE, Marshall WL. Advances in the treatment of adult incarcerated sex offenders. In: Beech AR, Craig LA, Browne KD (eds.) Assessment and treatment of sex offenders: a handbook. John Wiley & Sons Ltd; 2009. p. 329–47.

[56] Seto MC (2019). The motivation-facilitation model of sexual offending. Sex Abuse.

[57] Dennison SM, Stough C, Birgden A (2001). The big 5 dimensional personality approach to understanding child molesters. Psychol Crime Law.

[58] Hanson RK, Harris AJR (2000). Where should we intervene? Dynamic predictors of sex offense recidivism. Crim Justice Behav.

[59] Mann RE, Hanson RK, Thornton D (2010). Assessing risk for sexual recidivism: some proposals on the nature of psychologically meaningful risk factors. Sex Abuse.

[60] Boduszek D, Debowska A, Sherretts N, Willmott D, Boulton M, Kielkiewicz K (2019). Are prisoners more psychopathic than non-forensic populations? Profiling psychopathic traits among prisoners, community adults, university students, and adolescents. Deviant Behav.

[61] Porter S, Woodworth M. Psychopathy and aggression. In: Patrick CJ (ed.) Handbook of psychopathy. The Guilford Press; 2006. p. 481–94.

[62] DeLisi M, Peters DJ, Dansby T, Vaughn MG, Shook JJ, Hochstetler A (2014). Dynamics of psychopathy and moral disengagement in the etiology of crime. Youth Violence Juv Justice.

[63] Walters GD (2012). Psychopathy and crime: testing the incremental validity of PCL-R-measured psychopathy as a predictor of general and violent recidivism. Law Hum Behav.

[64] Carter AJ, Mann RE, Laws D, O’Donohue W (2016). The strengths of treatment for sexual offending. Treatment of child molesters.

[65] Mann RE (2004). Innovations in sex offender treatment. J Sex Aggress.

[66] Schmucker M, Lösel F (2017). Sexual offender treatment for reducing recidivism among convicted child molesters: a systematic review and meta-analysis. Campbell Syst Rev.

[67] Gilbert P, Davies J, Nagi C (2017). Exploring compassion focused therapy in forensic settings: an evolutionary and social-contextual approach. Individual psychological therapies in forensic settings: research and practice.

[68] Pozza A, Dettore D (2017). Drop-out and efficacy of group versus individual cognitive behavioural therapy: what works best for Obsessive-Compulsive Disorder? A systematic review and meta-analysis of direct comparisons. Psychiatry Res.

[69] First MB, Williams JBW, Karg RS, Spitzer RL (2017). Structured clinical interview for DSM-5 disorders, clinician version (SCID-5-CV).

[70] Bonta J, Harland AT (1996). Risk-needs assessment and treatment. Choosing correctional options that work: defining the demand and evaluating the supply.

[71] Gunst E, Watson JC, Desmet M, Willemsen J (2017). Affect regulation as a factor in child molesters. Aggress Violent Behav.

[72] Strickland J, Parry CL, Allan MM, Allan A (2017). Alexithymia among perpetrators of violent offences in Australia: implications for rehabilitation. Aust Psychol.

[73] Engel J, Veit M, Sinke C, Heitland I, Kneer J, Hillemacher T, Hartmann U, Kruger TH (2019). Same same but different: a clinical characterization of men with hypersexual disorder in the sex@ brain study. J Clin Med.

[74] Byrne G, Bogue J, Egan R, Lonergan E (2016). “Identifying and describing emotions” measuring the effectiveness of a brief, alexithymia-specific, intervention for a sex offender population. Sex Abuse.

[75] Baly A, Butler S (2017). Empathy deficits and adolescent sexual offending: a systematic review of the evidence base. Aggress Violent Behav.

[76] Gery I, Miljkovitch R, Berthoz S, Soussignan R (2009). Empathy and recognition of facial expressions of emotion in child molesters, non-child molesters and normal controls. Psychiatry Res.

[77] Day A, Casey S, Gerace A (2010). Interventions to improve empathy awareness in sexual and violent offenders: conceptual, empirical, and clinical issues. Aggress Violent Behav.

[78] Seto MC (2005). Is more better? Combining actuarial risk scales to predict recidivism among adult child molesters. Psychol Assess.

[79] Longenecker JM, Krueger RF, Sponheim SF (2020). Personality traits across the psychosis spectrum: a hierarchical taxonomy of psychopathology conceptualization of clinical symptomatology. Pers Ment Health.

[80] Pozza A, Coluccia A, Kato T, Gaetani M, Ferretti F (2019). The ‘Hikikomori’ syndrome: worldwide prevalence and co-occurring major psychiatric disorders: a systematic review and meta-analysis protocol. BMJ Open.

[81] Gualtieri G, Traverso S, Pozza A, Ferretti F, Carabellese F, Gusinu R, Coluccia A (2020). Clinical risk management in high-security forensic psychiatry residences. Protecting patients and health professionals: perspectives and critical issues of the Law 81/2014. La Clin Ter..

[82] Stemple L, Flores A, Meyer IH (2017). Sexual victimization perpetrated by women: federal data reveal surprising prevalence. Aggress Violent Behav.

[83] Shields RT, Cochran JC (2020). The gender gap in sex offender punishment. J Quant Criminol.

[84] Carabellese F, Urbano M, Carabellese F, Gualtieri G, Pozza A, Ferretti F, Coluccia A (2020). The judicial treatment of sex offenders: old limits, new opportunities. Ital J Criminol.

[85] Volkert J, Gablonski TC, Rabung S (2018). Prevalence of personality disorders in the general adult population in Western countries: systematic review and meta-analysis. Br J Psychiatry.

[86] Winsper C, Bilgin A, Thompson A, Marwaha S, Chanen AM, Singh SP (2020). The prevalence of personality disorders in the community: a global systematic review and meta-analysis. Br J Psychiatry.

[87] McNally RJ (2016). Can network analysis transform psychopathology?. Behav Res Ther.

[88] Cervin M, Perrin S, Olsson E, Aspvall K, Geller DA, Wilhelm S (2020). The centrality of doubting and checking in the network structure of obsessive–compulsive symptom dimensions in youth. J Am Acad Child Adolesc Psychiatry.

[89] Barcaccia B, Cervin M, Pozza A, Medvedev ON, Baiocco R, Pallini S (2020). Mindfulness, self-compassion and attachment: a network analysis of psychopathology symptoms in adolescents. Mindfulness.

[90] Coluccia A, Ferretti F, Fagiolini A, Pozza A (2015). Incidenza E fattori di rischio per disturbi psicotici nelle popolazioni migranti in Europa: Una meta-analisi di studi trasversali. Rassegna Italiana di Criminologia.

